# Development and Cultural Adaptation of Psychological First Aid for COVID-19 Frontline Workers in American Indian/Alaska Native Communities

**DOI:** 10.1007/s10935-022-00695-y

**Published:** 2022-07-16

**Authors:** Fiona Grubin, Tara L. Maudrie, Sophie Neuner, Maisie Conrad, Emma Waugh, Allison Barlow, Ashleigh Coser, Kyle Hill, Shardai Pioche, Emily E. Haroz, Victoria M. O’Keefe

**Affiliations:** 1 Department of International Health, Social and Behavioral Interventions, Center for American Indian Health, Johns Hopkins Bloomberg School of Public Health, 415 N. Washington St. 4th Floor, Baltimore, MD 21231 USA; 2 Tribal Community Behavioral Health, Tahlequah, USA; 3 Department of Indigenous Health, School of Medicine and Health Sciences, University of North Dakota, Grand Forks, USA

**Keywords:** Cultural adaptation, American Indian/Alaska Native, Psychological first aid, Indigenous, Mental health, COVID-19

## Abstract

The coronavirus disease 19 (COVID-19) pandemic is broadly affecting the mental health and well-being of people around the world, and disproportionately affecting some groups with already pre-existing health inequities. Two groups at greater risk of physical and/or mental health detriments from COVID-19 and more profoundly impacted by the pandemic include frontline workers and American Indian/Alaska Native (AI/AN) communities. To provide support and prevent long-term mental health problems, we culturally adapted a psychological first aid guide specifically for COVID-19 frontline workers serving AI/AN communities. We engaged a diverse, collaborative work group to steer the adaptation content and process. We also held two focus group discussions with frontline workers in AI/AN communities to incorporate their perspectives into the adapted guide. Results from the group discussions and the collaborative work group were compiled, analyzed to extract themes and suggestions, and integrated into the adapted content of the guide. Main adaptations included updating language (i.e., to be more culturally appropriate, less prescriptive, and less text heavy), framing the guide from a harm-reduction lens, incorporating cultural activities, values, and teachings common across diverse AI/AN communities (e.g., importance of being a good relative), and validating feelings and experiences of frontline workers. The resulting adapted guide includes four modules and is available as a free online training. Our adaptation process may serve as a guiding framework for future adaptations of similar resources for specific groups. The adapted guide may stand as an enduring resource to support mental well-being, the prevention of mental health problems, and reduction of health inequities during the pandemic and beyond.

## Introduction

Since the invasion and violent colonization of their ancestral homelands, American Indian and Alaska Native (AI/AN) communities have experienced devastating morbidity and mortality due to infectious diseases. The determinants that threaten AI/AN communities during infectious disease outbreaks today include concentrated poverty, multigenerational homes, lack of access to culturally appropriate health care, historical trauma, and food and water insecurity (Brave Heart & DeBruyn, 1998; Curtice & Choo, 2020). Cumulatively, these factors set the stage for striking inequities experienced by AI/AN communities during the COVID-19 pandemic. According to the Centers for Disease Control and Prevention (CDC), AI/ANs are significantly more likely to be hospitalized (3.2 times more likely) or die from COVID-19 (2.2 times more likely) compared to White, non-Hispanic persons (CDC, 2020a). Underlying these numbers is a data crisis minimizing the actual burden of COVID-19 morbidity and mortality and impacting allocation of resources to help mitigate and protect AI/AN communities (Howard-Bobiwash et al., 2021; UIHI, 2021). COVID-19-related mortality disproportionately affects those aged 65 and older (CDC, 2020b); in AI/AN communities, the loss of Elders who are revered in Tribal cultures as keepers of language and Indigenous Traditional Knowledges (ITK; Gone, 2012) will leave devastating impacts on communities for generations (Healy & Blue, 2021).

Prior to the pandemic AI/ANs experienced disproportionately higher rates of some mental health disorders, including higher rates of anxiety and trauma-related disorders, suicide and violence, and binge substance use (Brave Heart & DeBruyn, 1998; Gone & Trimble, 2012; SAMHSA, 2018). These inequities are driven by historical trauma (Brave Heart & DeBruyn, 1998) and social determinants resulting from settler colonialism and persistent structural racism. Importantly, there are more than 574 federally recognized tribes in the United States and many urban AI/AN communities, and mental health inequities vary widely within and across contexts (SAMHSA, 2018).

Access to mental health services also varies broadly. Before the COVID-19 pandemic, hundreds of Indian Health Service (IHS), tribal, and urban AI/AN health facilities reported staff shortages, including mental health providers, which limits access to mental health services for AI/AN people (HHS, 2011). Even when mental health services are available, AI/AN individuals often face physical barriers (e.g., distance to clinic, transportation), social barriers (e.g., not having childcare, stigma, and lack of confidentiality in overcrowded clinics serving tight-knit communities), or economic barriers (e.g., challenges to paying copays; HHS, 2011). In addition, there is no guarantee that providers are trained to understand tribal worldviews or that clients have access to culturally appropriate services during treatment and recovery (SAMHSA, 2018). Practicing and reclaiming cultural traditions are an important part of prevention, recovery, and overall wellness for AI/ANs (SAMHSA, 2018).

Early evidence suggests the COVID-19 pandemic has had a significant and immediate impact on mental health globally (Cullen et al., 2020). Much like disparities in morbidity and mortality due to the virus itself, the pandemic has increased national attention on mental health inequities, as racial and ethnic minority groups continue to experience a disproportionate burden of mental health impacts related to the pandemic (Ettman et al., 2020; Saltzman et al., 2021). In AI/AN communities, the mental, emotional, and spiritual health impacts of COVID-19 are expected to be long lasting given the immense and cumulative losses experienced during the pandemic (Arrazola et al., 2020). In a Canadian survey of 1400 Indigenous youth and adults collected in April and May of 2020, almost two-thirds of participants reported that their mental health had become “somewhat worse” or “much worse” since physical distancing began (Arriagada et al., 2020). At this point in time, interventions that aim to prevent long-term mental health problems from COVID-19 are imperative for strongly impacted AI/AN communities.

The mental health impacts of the COVID-19 pandemic may also disproportionately affect frontline workers. Frontline workers are a subgroup of essential workers (i.e., people whose jobs involve work that is deemed necessary to maintain vital aspects of society) distinguished by the characteristic that the work they do cannot feasibly be performed remotely (e.g., health care workers, grocery store employees, truck drivers; Blau et al., 2021). The mental well-being of frontline workers is a critical component of the pandemic response, especially among those in health care (WHO, 2020). Health care workers responding to past outbreaks of infectious diseases experienced burnout and detriments to their mental health, which continued even after the outbreaks had been contained (Shah et al., 2020). The COVID-19 pandemic perpetuates this trend. Frontline health care workers report increased depression and psychological distress, deterioration in sleep quality, and have been labeled one of the highest risk groups for developing psychiatric symptoms during COVID-19 (Vindegaard & Benros, 2020). Increase in burnout among frontline healthcare workers during the COVID-19 pandemic is concerning, as it is not only associated with detriments to individual well-being, but can also decrease quality of patient care and healthcare system function (Patel et al., 2018). Additionally, frontline workers have families and often children of their own to care for, all of whom may be subsequently affected by the challenges and mental health stressors frontline workers experience.

There is an urgent need for targeted psychosocial interventions to address burnout and distress prevention among frontline workers during the COVID-19 pandemic (Cullen et al., 2020; Shah et al., 2020). Psychological first aid (PFA), as defined by the World Health Organization, is a “humane, supportive response to a fellow human being who is suffering and who may need support” that may provide immediate support to individuals experiencing distress due to a recent crisis such as COVID-19 (Minihan et al., 2020). PFA has been adapted for use in the context of numerous disasters, different populations, and regions around the world (Sim & Wang, 2021), however, a lack of empirical data evaluating the effectiveness of PFA interventions persists, in part due to the fact that PFA is intended to be implemented in the aftermath of a disaster when carefully planning an evaluation study is less feasible and urgent (Fox et al., 2012). Despite the lack of robust evidence to support PFA’s effectiveness, many sources corroborate PFA as an “evidence-informed intervention,” and experts generally agree that it is a useful and worthwhile tool of which to pursue future implementation and outcome evaluation (Fox et al., 2012; McCabe et al., 2014; Shultz & Forbes, 2013; Sim & Wang 2021).

A more recent assessment uncovered preliminary evidence that PFA training can significantly impact knowledge of PFA skills and subsequent self-efficacy to employ those skills to foster resilience and coping (Wang et al., 2021). A review of interventions to support mental health of frontline workers during and after infectious disease outbreaks found a general lack of evidence for such interventions (i.e., evidence currently does support any alternatives that would be better than PFA; Pollock et al., 2020), and given PFA’s relatively low cost and history of successful implementation in such settings, its continued application is warranted (Minihan et al., 2020). PFA is also a tool that can be provided by anyone, regardless of their training, making it particularly suitable for Tribal communities with limited access to mental health care work forces. PFA is designed to provide those responding to a traumatic event with skills and resources necessary to reduce their own and others’ distress in the short-term and develop positive coping strategies to avoid prolonged mental health problems (Birkhead & Vermeulen, 2018). Several clinicians concur that PFA is “essential” for “addressing stress-related reactions after traumatic events like the COVID-19 pandemic.” (Shah et al., 2020).

To address the need for culturally relevant mental health support in AI/AN communities and for frontline workers (BlackDeer & Silver Wolf, 2020; Muller et al., 2020), we adapted a PFA intervention to create a needed resource for COVID-19 frontline workers in AI/AN communities. We adapted this resource to improve its acceptability and relevance in line with recommendations by other researchers to address social/structural health determinants when culturally adapting prevention approaches (Greenfield et al., 2013). The purpose of this paper is to describe the adaptation process and results. This cultural adaptation of a PFA intervention for AI/AN communities may offer novel insights that could benefit future endeavors to adapt similar interventions.

## Methods

### The Intervention

The Inter-Agency Standing Committee Reference Group on Mental Health and Psychosocial Support in Emergency Settings (IASC MHPSS RG) created “Basic Psychosocial Skills–A Guide for COVID-19 Responders” to assist orientation and integration of psychosocial support for COVID-19 frontline workers across the globe in May 2020 (IASC, 2020). The IASC guide was created with input from two consultation sessions with members of the IASC MHPSS RG and refined with survey feedback from survivors of and responders to the COVID-19 pandemic globally (IASC, 2020). The guide contains five modules as well as annexes with additional resources such as space to organize managerial roles, log daily routines, and more (IASC, 2020). Two online trainings including videos, quizzes, and case examples have been created from the IASC guide, published as adaptations and are available for free online (The Asia Foundation et al., 2020; UTS, 2020).

### Guiding Frameworks

Our cultural adaptation of the IASC guide was informed by an Indigenous framework, Walters and Simoni’s (2002) Indigenist Stress-Coping Model. The Indigenist Stress-Coping Model is grounded in empirical evidence that shows historical trauma, as well as other contemporary forms of discrimination, influence AI/AN peoples’ physical and mental health (Walters & Simoni, 2002). Despite facing many physical, mental, and emotional stressors, AI/AN peoples are resilient and cultural buffers (e.g., identity attitudes, spiritual coping, enculturation, traditional health practices) can protect against the negative impacts of stress (Walters & Simoni, 2002). Building on this framework, our adaptation process focused on engaging with culture and community as protective measures against stress, as well as using cultural values to promote positive mental health behaviors.

Previous work assessing the evidence base for PFA notes that the applications and adaptations of PFA vary, with more standardization needed to support evaluation of PFA interventions (Wang et al., 2021). To help bring more standardized structure to our adaptation of this PFA resource, we referenced McCabe et al.’s (2014) model of core competencies of PFA developed collaboratively among the US CDC and various schools of public health. This model outlines knowledge, skills, and attitudes related to six domains of core competencies: (1) initial contact, rapport building, and stabilization; (2) brief assessment and triage; (3) intervention; (4) triage; (5) referral, liaison, and advocacy; and (6) self-awareness and self-care (McCabe et al., 2014). We reviewed these competencies and noted which parts of our adapted PFA resource aligned with each, ensuring that all core competency domains were incorporated into the content of the adapted resource. We also integrated information and suggestions from the WHO’s (2020) guidance on mental health and psychosocial considerations during COVID-19 into the final resource.

### Cultural Adaptation Process

We engaged a collaborative work group (CWG) of AI/AN experts to steer cultural revisions and adaptations of the original IASC guide. Additionally, we held two focus group discussions (FGDs) to gather input from frontline workers serving AI/AN communities to ensure the content of the guide was relevant and responsive to their needs, concerns, and experiences. After revising and refining the adapted content, we worked with an Indigenous-owned graphic design firm to create the adapted guide and online training with a culturally relevant, cohesive design. We use the Framework for Reporting Adaptations and Modifications-Expanded (FRAME) to specify and report adaptations and modifications to the PFA guide for frontline workers in AI/AN communities in a systematic way (Wiltsey Stirman et al., 2019). An overview of the adaptations following the FRAME framework is available in Table 3.

### Data Collection

#### Collaborative Work Group

Our approach to this project was informed by community-based participatory research (CBPR), a way of conducting research that considers inequities at the social, structural, physical, and environmental levels and includes actively involving community members and other important stakeholders in all phases of a project (Israel et al., 2001). We incorporated CBPR principles specific to working with AI/AN communities, such as recognition of tribal sovereignty, interpreting data within the cultural context, and utilizing Indigenous ways of knowing (Laveaux & Christopher, 2009). As part of our CBPR approach, to inform the cultural adaptation of the PFA guide, we convened a collaborative work group (CWG) comprised of six AI/AN people and one ally, including two psychologists, the executive director of an AI/AN urban health clinic, one IHS hospital nurse, one IHS pharmacy director, one physician/public health professional, and one public health/mental health professional. All CWG members were purposively recruited due to their work in reservation and urban AI/AN communities and were invited to steer the project, give specific input as to what content should be included, and guide implementation of adaptations. The project team and CWG met eight times virtually via video conference in September-November 2020 to develop and refine adaptations to the PFA guide.

First, CWG members independently reviewed the original IASC PFA guide. In the first meeting, we gathered overall impressions of the IASC guide to assess whether it represented a helpful and feasible resource to adapt and perspectives on mental health needs among frontline workers in AI/AN communities. In subsequent meetings, the research team presented each module of the IASC PFA guide to obtain CWG feedback as to what existing components of the original guide should be included, removed, changed, or what new content should be added. In keeping with a CBPR-informed approach, we invited all CWG members to co-author this manuscript to honor their substantial intellectual contributions to this work.

#### Focus Group Discussions

We conducted two focus group discussions (FGDs) with COVID-19 frontline workers in AI/AN communities. One focus group included seven frontline workers from the Southwest US, and the second included two frontline workers in urban AI/AN communities in the Northeast and Midwest US. Both focus group discussions took place virtually using Zoom with a facilitator and notetaker. Participants were asked to reflect on the collective experience of frontline workers responding to COVID-19 in their communities. The Institutional Review Board (IRB) at the Johns Hopkins Bloomberg School of Public Health (BSPH) determined this project to be non-human subjects research that did not require oversight by the IRB.

FGDs were facilitated by a member of the research team who had no existing personal relationship with participants. FGD participants had not read the IASC PFA guide and were not affiliated with the CWG. The facilitator used a guide to ask questions about: (1) mental and behavioral health concerns, challenges, and stressors; (2) coping skills and existing supports/resources; and (3) resources needed or that could be improved. The first FGD took place in December 2020 and the second in January 2021.

### Data Analysis

After each CWG meeting, the project team incorporated their feedback into draft adaptations of the guide and then presented it back to the CWG in subsequent meetings. This process allowed for checking the completeness and validity of incorporated suggestions and was used to iteratively revise and refine content. When CWG members were unable to attend meetings, they provided written feedback via email.

Using notes and transcripts form the FGDs, the research team used a qualitative content analysis approach (Hsieh & Shannon, 2005) to determine which ideas could be appropriately addressed within the scope of the adapted PFA guide. Three researchers carefully read and re-read the data to extract and organize quotes into a spreadsheet, grouping quotes reflecting similar concepts and ideas together. A fourth project team member reviewed the coded data to prioritize which ideas and concepts should be added to the adapted guide content.

## Results

### CWG Results

Input and guidance from the CWG across eight meetings were compiled, reviewed, and incorporated to create the adapted content. Results are organized into (1) General input and (2) Specific feedback on the original guide.

#### General Input on Needs, Concerns, and Resources for Frontline Workers

At the outset, CWG members appreciated the original IASC guide’s note that first responders comprise a diverse group of people (e.g., health professionals, law enforcement, food service workers) and emphasized that frontline workers include more than just health care workers (e.g., grocery workers, sanitation workers) and that taking into consideration this entire audience would be important for developing an effective resource. When asked about mental health concerns among frontline workers, CWG members described anxiety (e.g., related to contracting and spreading COVID-19 to family and friends), feeling overburdened (e.g., taking on additional responsibilities while their normal job duties have intensified), difficulty balancing professional and personal responsibilities (e.g., staying home to support children in virtual learning), social and cultural isolation (e.g., feeling lonely, missing gathering or traditional community activities), and feeling frustrated and confused by the rapid spread of COVID-19 misinformation.

When asked what strategies and resources were already available and useful for frontline workers, CWG members mentioned tele-behavioral health services, engaging in cultural activities, and spending time outside. CWG members noted that some organizations were not adapting their policies quickly enough to align with the context of the pandemic and that policy updates were needed to increase flexibility and paid time off for frontline workers.

#### Specific Input on the Original Guide

CWG members generally agreed the guide was a helpful, relevant, and appropriate resource to adapt. The CWG recommended incorporating an overall theme of Indigenous relationality to highlight the importance of caring for all our relations, which includes maintaining balanced relationships among physical, spiritual, and mental health, as well as culture, family, and community. This also involves caring for AI/AN communities across diverse settings (e.g., rural, reservation-based, urban). Specific CWG recommendations included: (1) to take a strengths-based approach; (2) to validate that people may feel additional stress from historical trauma related to previous experiences with epidemics among AI/AN communities; (3) to promote a sense of relationality and connectedness to combat feelings of social and cultural isolation; and (4) to update and include relevant case examples to reflect AI/AN tribal and urban frontline worker experiences as helpful examples of how to apply the resources, skills, and knowledge contained in the guide. See Table 1 for a summary of CWG feedback and suggested adaptations organized by modules of the IASC guide.

**Table 1:** Summary of CWG guidance incorporated into adapted guide^a^

Module (from original guide)	General feedback on IASC guide	General input on how to adapt the IASC guide
Module 1: Your well-being	Dislike language framing COVID-19 response as a responsibility. This could increase stress, and some people did not sign up for this responsibility.	Include the importance of taking care of yourself as it relates to taking care of others.
	Note about feeling overburdened rings true, this can be expanded on. People may feel they have new responsibilities and cannot deliver on them all.	Highlight importance of being a good relative.
	Instructing people to stay up-to-date on information feels patronizing.	Validate feelings of inadequacy, fear, anxiety.
	Suggestions around adhering to a schedule and minimizing substance use feel patronizing.	Acknowledge the sacrifices frontline workers are making and the impacts on them and their families.
	Keep the circles of control exercise.	Use a harm reduction approach.
	Case example of community leader is not helpful and should be removed.	Promote practicing gratitude.
		Incorporate traditional activities in suggestions for relaxing/coping (e.g., spiritual/prayer activities, appropriate use of traditional medicines).
		Reduce text heaviness by eliminating irrelevant examples.
		Use videos to engage people more.
Module 2: Supportive communication in everyday interactions	Not all body language is appropriate across Native communities (e.g., direct eye contact is sometimes considered disrespectful).	Include ideas for creating comfortable environments for virtual interactions, rapport building.
	Do’s and Don’ts chart is stressful.	Focus on empathy and love for all relatives.
	Active listening graphics should be reframed to highlight common cultural teachings around the importance of listening.	Highlight importance of listening as a cultural teaching.
		Incorporate humor.
Module 3: Offering practical support	Needs in addition to original list are mental health, cultural and spiritual needs, addressing misinformation.	Include information on how to support people in acute mental health crisis.
	Language about “manage their own problems” is negative.	Focus on caring for community and Elders as a positive action.
	Keep the case example.	Adapt language of stop-think-go tool to be more culturally relevant (changed to Stop-Reflect-Skoden) and succinct.
Module 4: Supporting people who are experiencing stress	Signs of stress ring true.	For descriptions of serious distress, add the following signs/symptoms: difficulty concentrating, difficulty sleeping, and binge behaviors. Remove the sign/symptom of a person not knowing their own name (i.e., orientation to person).
	Keep the deep breathing and progressive muscle relaxation exercises.	
Module 5: Helping in specific situations	Dislike language describing community members as “vulnerable” or “marginalized.”	Change language to “priority” instead of “vulnerable/marginalized.”
		Include the following groups of priority community members: (a) those who are unhoused; (b) people who are unsafe in their homes (e.g., because of domestic violence); (c) people who struggle with substance use; (d) people who live alone or away from home community; (e) people with pre-existing health or mental health conditions; and (f) Elders.

^a^A﻿ll of the feedback and suggestions described in this table were incorporated into the final, adapted guide. General feedback on the IASC guide refers to specific things about the IASC guide that the CWG noted should be changed, kept, or removed when adapting the materials. General input on how to adapt the IASC guide refers to guidance the CWG shared as to how to best change and adapt the IASC guide.

### FGD Results

FGD participants shared many recommendations made by the CWG with some additions. Participants shared that challenges facing frontline workers in AI/AN communities included burnout, difficulty balancing pre-pandemic roles with new duties and responsibilities (including being unable to work remotely like others), increased workload, fear of contracting and spreading COVID-19 to family, compassion fatigue, anger and frustration (coupled with a lack of outlets to share these feelings with someone who would understand), and missing traditional cultural and spiritual practices. Many participants also touched on technical challenges of physical distancing and not being able to connect with family and friends who live far away, often related to challenges with using virtual meeting technology, primarily due to lack of access to broadband internet.

When asked what resources or strategies could help frontline workers cope with these challenges, participants shared focusing on the positive successes of their work, harnessing community strengths, engaging in physical activity, spending time outside, connecting with religion/spirituality, finding safe ways to connect with family and friends, listening to music, and finding ways to safely engage in tribal and spiritual wellness practices. Participants also noted that the large amount of information from trusted sources was sometimes difficult to navigate and that more guidance around how to access and make use of this information is needed.

When discussing resources needed to support frontline workers, participants shared that more resources to support self-care practices among frontline workers are needed, and that supervisors should model this behavior. Participants also shared that children, especially those related to frontline workers, could use more support in coping with stress related to virtual school and being unable to visit in person with friends and extended family.

### Guide Structure and Content

**Fig. 1:**
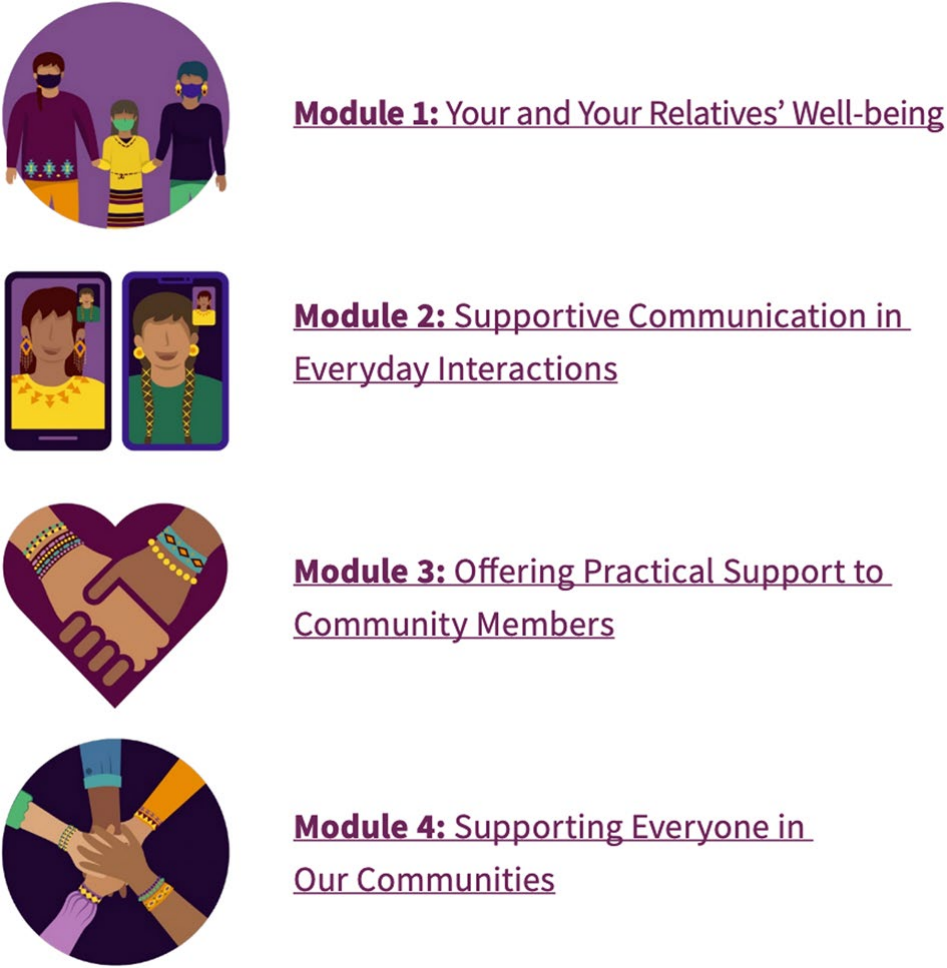
displays the table of contents page showing the four main modules from the adapted psychological first aid for COVID-19 frontline workers in AI/AN communities guide. Descriptive caption for accessibility: four lines of text are displayed next for four colorful images; each line and image represent a module in the table of contents of the adapted PFA guide. The first line says, “Module 1: Your and Your Relatives’ Well-being” and includes an image of three people together holding hands. The second line says, “Module 2: Supportive Communication in Everyday Interactions” and is next to an image of two people using virtual video call on cell phones. The third line says, “Module 3: Offering Practical Support to Community Members” and is next to an image of two hands holding each other in a heart shape. The last line says, “Module 4: Supporting Everyone in Our Communities” and is next to a circular image of five hands placed on top of each other

The final PFA for Frontline Workers in AI/AN Communities guide comprises four main modules (see Fig. 1; Table 2). The original guide’s first and fourth modules were combined in response to suggestions to reduce text heaviness and make more explicit the connection between self-care and caring for others. To incorporate all feedback and recommendations while maintaining a succinct, easy-to-use guide, we included five additional annexes with specific resources for supervisors/managers (adapted from the IASC guide annex on this topic), information on burnout and compassion fatigue (developed from FGD input), relaxation exercises (adapted from the IASC guide’s annex on progressive muscle relaxation), supporting children (developed from FGD input), and guidance on trusted COVID-19 resources and how to access them (developed from CWG input).

**Table 2:** Adaptations to basic psychosocial skills: a guide for COVID-19 responders

Program component	Basic psychosocial skills: a guide for COVID-19 responders (original guide)	Psychological first aid for COVID-19 frontline workers in AI/AN communities (adapted guide)
Guide contents		
Modules	1. Your well-being	1. Your and your relatives’ well-being
	2. Supportive communication in everyday interactions	2. Supportive communication in everyday interactions
	3. Offering practical support	3. Offering practical support to community members
	4. Supporting people who are experiencing stress	4. Supporting everyone in our communities
	5. Helping in specific situations	
Annexes	A. Advice for managers and supervisors who support staff and volunteers	A. Resources for supervisors & managers
	B. Daily routine schedule	B. Burnout & compassion fatigue
	C. Circles of control	C. Relaxation exercises
	D. Progressive muscle relaxation instructions	D. Supporting children
	E. Resources in your area	E. Trusted COVID-19 resources
Case examples	1. Patrick’s case: community leader	1. Case example: Coping with stress and misinformation
	2. David’s case: communication	2. Case example: Caring for elders who need access to food or other essential items
	3. Asma’s case: supportive listening	
	4. Precious’ case: supporting people who are grieving	3. Case example: Tamara and Connor use Stop-Reflect-Skoden!
	5. Priya and Deepak’s case: supporting elders	
	6. Ahsan and Mohammad’s case: Stop-Think-Go	
	7. Josephine and Julia’s case: staying connected virtually	
Training	Online	Online
	Free	Free
	No registration required	Registration required
	Asynchronous	Asynchronous
	Knowledge assessed via quizzes hosted on google forms	Knowledge assessed via quizzes in online learning platform

In line with CWG guidance, four case examples demonstrate how to apply resources and knowledge contained in the guide and represent experiences that may be familiar to frontline workers in AI/AN communities. The case examples in the guide include: (1) Caring for Elders: an applied example of how to stay connected while keeping Elders safe, as well as the importance and benefits of doing so; (2) Coping with stress and misinformation: an example that validates the stress frontline workers experience and offers practical solutions to cope; and (3) Stop-Reflect-Skoden: an example of how to apply the adapted problem solving tool from the guide. The annex includes a case example about supporting workers who are adapting to remote work environments.

Motivated by two examples of the IASC guide turned into online trainings, we also created an online training to accompany this specific adaptation. The online training brings the guide’s content to life to teach skills and concepts. To enhance continuity across the guide and training, the same Indigenous-owned design firm developed videos for the training. Videos in the course include AI voice actors who deliver lectures and narrate case examples. Content is delivered in eight short videos including an introduction, four modules, and three case examples. The online training is hosted for free on CoursePlus, a learning platform at BSPH, and any interested person may register and complete the training and receive a certificate of completion. Table 3 summarizes the final adaptation results.

**Table 3:** Summary of adaptations/modifications organized by FRAME^a^ categories

Frame category	Descriptions of adaptations/modifications
1. When did the modification occur?	Pre-implementation/dissemination of this resource
2. Were the adaptations planned?	Planned, reactive (in response to the COVID-19 pandemic)
3. Who participated in the decision to modify?	Researchers, public health professionals, health care workers, community members, funder
4. What was the goal?	Improve fit with recipients to make the resource culturally relevant and appropriate
5. What is modified?	*Content*: text and visual content of the resource
	*Training and evaluation*: changed the platform that delivers the training and designed an evaluation study specifically for this resource
	*Dissemination*: promoted through social media, sharing with nationwide organizations in the US
6. At what level of delivery (for whom/what) is the modification made?	*Community*: AI/AN communities in the US
	*Target group*: frontline workers
7. What is the nature of content modification?	*Refining*: adjusting some existing language and content
	*Adding elements*: adding new content
	*Removing elements*: removing irrelevant or excess content
	*Shortening/condensing*: overall making the guide shorter and less text heavy
	*Reordering of modules/segments*: combined two modules that overlapped significantly
8. Relationship fidelity/core elements?	Fidelity consistent, core elements and functions (i.e., to support coping, resource sharing, and mental well-being and prevent long-term mental health problems from developing) preserved

^a^Categories of the FRAME framework come from Wiltsey Stirman et al. (2019)

### Implementation and Expected Outcomes

The final guide and annexes are publicly available and downloadable at http://bit.ly/PsychFirstAid4COVID19. Instructions for how to access and navigate the free online training, along with information about this resource and how it was created, are also posted on this webpage. As the resource was created broadly to support frontline workers in AI/AN communities, it is widely available online for individuals to access and use, or for organizations to incorporate as part of their own training. In an effort to spur uptake of this resource by making it more worthwhile and beneficial, the training course was approved for *AMA PRA Category 1 Credits™* between August 2021 and May 2022 so that health care and other professionals could obtain continuing education credit for completing the online training course.

While the final, adapted PFA resources are specifically intended to support frontline workers in AI/AN communities by providing them with the knowledge, skills, and attitudes to support mental well-being, ultimately, we hope and envision that they will have a wider impact. When frontline workers are equipped with the skills to support psychosocial well-being, those around them (e.g., patients, family) may also experience positive effects. In the short-term, this may look like reduced levels of acute stress and increased coping behaviors, and in the long-term the intended impact is the prevention of mental health problems from developing or worsening as a result of the pandemic and related mental health stress. Additionally, for many Indigenous communities, well-being (including mental well-being) of individuals and families is inherently connected to holistic community health (Ullrich, 2019). Within Indigenous communities, mental health is interconnected with physical health, emotional health, and spiritual health, and is tied to relationships at multiple levels (O’Keefe et al., 2022). From this understanding, when networks of individuals and community members are supported with this PFA resource, it could contribute to wider well-being for entire communities.

## Discussion

AI/AN communities are resilient and working diligently to address mental health concerns during the pandemic through culturally congruent approaches grounded in local values and Tribal sovereignty. COVID-19 has accelerated the expansion of telehealth services available through IHS, addressing critical gaps in mental health care (IHS, 2021). Organizations such as the Urban Indian Health Institute hold policymakers accountable for the lack of data on COVID-19 in AI/AN populations (UIHI, 2021). Importantly, AI/AN people are creatively celebrating their culture while protecting their communities through social distancing (PBS, 2021). From virtual pow wows to online tribal language classes and storytelling, AI/AN communities harness strengths and cultural values that have supported Indigenous health and wellness since time immemorial. Nonetheless, the need for culturally appropriate mental health support in AI/AN to communities remains, as does the need for interventions that help prevent mental health problems from developing or worsening as a result of the COVID-19 crisis. We culturally adapted the PFA and training tools to support the psychosocial well-being of frontline workers serving AI/AN communities, a group at the intersection of higher risk for COVID-19 exposure and subsequent mental health impacts (CDC, 2020a; Vindegaard & Benros 2020).

As the COVID-19 pandemic evolves, this adapted resource and the process used to create it continue to be useful. The core competencies of PFA (e.g., self-care, referral, advocacy) remain relevant for many frontline workers, and this adaptation process could be used in the future to create PFA resources for new disaster events or other interventions. We report our process for adapting this guide with structure and transparency to (1) respond to calls to increase standardization of reporting adaptations of prevention interventions so as to better support implementation (Chambers & Norton, 2016); and (2) to facilitate future adaptation processes, as AI/AN communities may benefit from similarly adapted resources. Specifically, other adaptations of PFA resources could be useful to support psychosocial well-being during other disaster events that impact AI/AN communities, such as fires, earthquakes, or floods (Standing Horse, 2017). PFA interventions may be particularly suitable for AI/AN communities because they help train non-specialist providers to support the community, an important consideration given the lack of mental health providers in AI/AN communities (O’Keefe et al., 2021; Standing Horse, 2017). Many AI/AN community members are well-placed to offer mental health support during times of crisis, and PFA interventions can help provide a common framework to broaden their reach while elevating valuable cultural knowledge and teachings to best serve the community.

Critically, implementation of the adapted PFA guide is now being studied to learn about its impact on frontline workers’ anxiety, coping, burnout, self-efficacy, communal mastery, stress, and positive mental health. The evaluation includes a pre/post assessment before and after the training to determine (1) whether frontline workers experience significant and lasting impacts on frontline worker mental health, knowledge, and competencies after completing the training course; and (2) whether the resource is generally perceived as useful and relevant. While PFA is widely disseminated, the evidence-base for this type of intervention is limited (Fox et al., 2012; Shultz & Forbes, 2013), with no previous studies being done in partnership with Indigenous communities. It will also be ultimately important to understand the cost-effectiveness of these approaches given the relative ease of disseminating and delivering PFA resources in contexts of disaster events. By evaluating this resource while the COVID-19 pandemic is ongoing, we can contribute valuable, urgently needed data to the developing evidence base for PFA (Fox et al., 2012).

Future work should also consider the mental and behavioral health impacts the pandemic has on children and families of frontline healthcare workers. Frontline healthcare workers do not experience the psychosocial stressors of the pandemic alone, and their children and families may need specific support during and after the pandemic (Souadka et al., 2020). Research and resources are needed to support the unique mental health and well-being needs of children of frontline healthcare workers.

## Limitations

It is difficult, if not impossible, to balance the breadth of cultural diversity across all Tribal Nations when creating a broad resource intended for use across these communities. Thus, the adapted guide may be missing important perspectives, and its cultural congruency may vary across tribal communities. However, we engaged a diverse CWG to steer the cultural adaptation, with specific attention to inclusivity of urban, rural, and reservation communities, and we hope these efforts have resulted in a resource that is widely applicable and useful in AI/AN communities. Another limitation of this adaptation process is that it is not culturally grounded (i.e., developed directly by and for a community and centering the sociocultural context of a specific target population; Okamoto et al., 2014) but rather posited that the underlying core components of the original resource are widely applicable across AI/AN communities. While culturally grounded approaches often result in stronger, more robust interventions, they can be significantly more time consuming to develop (Okamoto et al., 2014), and in the context of the COVID-19 crisis we felt time pressure to produce this resource expediently. Finally, a potential limitation when adapting any intervention is the tradeoff between maintaining fidelity to the original program while making changes during adaptation (Chambers & Norton, 2016). We have attempted to minimize this limitation and retain fidelity to key elements of PFA by incorporating standardized core components of PFA (McCabe et al., 2014) and reporting our adaptation process using the FRAME framework (Wiltsey Stirman et al., 2019) to provide clear information about modifications, reasons for modification, and fidelity to the original resource. This approach aligns with best practices for striving to achieve fidelity when adapting evidence-based programs (Martinez et al., 2019).

## Conclusions

The COVID-19 pandemic has underscored existing health inequities around the world, including the pandemic’s disproportionate impact on AI/AN communities (Arrazola et al., 2020). The aim of this project was to promote health equity and prevent further health disparities by fostering culturally appropriate psychosocial support resources for frontline workers serving AI/AN communities. Inequity surrounding mental health in AI/AN communities is not a new phenomenon (Gone & Trimble, 2012), as such, this guide may continue to support mental health, health equity, and prevention of mental health problems beyond the pandemic. The process used to develop this guide may also serve as a framework by which to create similar, culturally adapted interventions in the future.
